# Gene expression profiling of lymphoblastoid cell lines from monozygotic twins discordant in severity of autism reveals differential regulation of neurologically relevant genes

**DOI:** 10.1186/1471-2164-7-118

**Published:** 2006-05-18

**Authors:** Valerie W Hu, Bryan C Frank, Shannon Heine, Norman H Lee, John Quackenbush

**Affiliations:** 1 The George Washington University Medical Center, Dept. of Biochemistry and Molecular Biology, 2300 Eye St., N.W. Washington, DC 20037, USA; 2 The Institute for Genomic Research, 9715 Medical Center Drive, Rockville, MD 20850, USA; 3 The Dana-Farber Cancer Institute, Department of Biostatistics and Computational Biology, 44 Binney St. Boston, MA 02115, USA

## Abstract

**Background:**

The autism spectrum encompasses a set of complex multigenic developmental disorders that severely impact the development of language, non-verbal communication, and social skills, and are associated with odd, stereotyped, repetitive behavior and restricted interests. To date, diagnosis of these neurologically based disorders relies predominantly upon behavioral observations often prompted by delayed speech or aberrant behavior, and there are no known genes that can serve as definitive biomarkers for the disorders.

**Results:**

Here we demonstrate, for the first time, that lymphoblastoid cell lines from monozygotic twins discordant with respect to severity of autism and/or language impairment exhibit differential gene expression patterns on DNA microarrays. Furthermore, we show that genes important to the development, structure, and/or function of the nervous system are among the most differentially expressed genes, and that many of these genes map closely *in silico *to chromosomal regions containing previously reported autism candidate genes or quantitative trait loci.

**Conclusion:**

Our results provide evidence that novel candidate genes for autism may be differentially expressed in lymphoid cell lines from individuals with autism spectrum disorders. This finding further suggests the possibility of developing a molecular screen for autism based on expressed biomarkers in peripheral blood lymphocytes, an easily accessible tissue. In addition, gene networks are identified that may play a role in the pathophysiology of autism.

## Background

Autism and related autism spectrum disorders (including Asperger's Syndrome and pervasive developmental disorder-not otherwise specified (PDD-NOS)) are considered to be among the most devastating psychiatric illnesses affecting children. The three core symptoms of autism spectrum disorders (ASD) are: 1) deficits in social interactions and understanding, 2) aberrant communication and/or language development, and 3) restricted interests and repetitive, stereotyped behaviors [1]. To date, there are no definitive molecular or genetic markers that allow unequivocal diagnosis of ASD, with the exceptions of tuberous sclerosis, Rett's Syndrome, and Fragile X Syndrome [2-12]. Together, these genetically defined mutations are present in only a minority of individuals (<10%) within the broad autism spectrum. The majority of diagnoses are dependent on behavioral characteristics, according to DSM-IV guidelines, using questionnaires such as the Autism Diagnostic Interview-Revised (ADI-R) [13] or the Autism Diagnostic Observation Schedule (ADOS) [14], which are structured to evaluate children who are approximately 2 or older in mental age. Although the guidelines are relatively clear, the individual rater's (eg., parents, teachers, clinicians, therapists) perception of the evaluated behavior leaves much room for ambiguity. Moreover, with the more mildly affected individuals (eg., with Asperger's Syndrome), diagnosis is often not made until well after the child starts school and, even then, the child is often diagnosed with other more common disorders (such as attention deficit disorder or learning disability) before Asperger's Syndrome is considered, which delays appropriate intervention and effective educational programming. Thus, there is a great need to identify biomarkers that can be used consistently in a clinical setting to diagnose ASD. Furthermore, it is important to identify biological processes that are associated with distinct ASD phenotypes in order to design effective drug therapies targeted to specific individuals.

Although genetic linkage analyses have identified numerous candidate genes for autism [15], there is little consistent data that would support the use of any (or a combination) of these as diagnostic biomarkers for ASD. Furthermore, each candidate gene alone lends little insight into the pathophysiology of these disorders, which are believed to arise from dysregulation of multiple genes. Recently, attention has turned to transcriptional profiling approaches [16-19], which involve simultaneous, large-scale expression analysis of thousands of genes on a cDNA (or oligonucleotide) microarray slide, to unravel complex psychiatric disorders. The advantage of transcriptional profiling using microarrays is the ability to study multiple genes in the context of functional gene networks within a living cell, as opposed to forward genetic approaches.

So far, application of microarrays to the study of autism has been described in just one study on post-mortem brain tissue from autistic subjects and matched tissue controls [20]. Thirty genes were identified as being differentially expressed in autistic brain samples relative to matched tissue controls on a combination of 2 separate array platforms containing 588 or 9374 cDNA probes, indicating that autism is associated with multiple disturbances in gene expression. Of this list, only a few genes related specifically to neurological functions and, of these, the glutamate receptor system was targeted for further study. In a similar vein, a recent bioinformatics analysis of autism positional candidate genes using biological databases and computational gene network prediction software demonstrates that the often disparate results from genetic studies implicating a multitude of different genes can be coalesced into interconnected but distinct pathways centered on a specific gene or genes (e.g., FOS and TP53), or on a particular biological theme, e.g., apoptosis [15]. Both of these studies suggest the involvement of multiple genes not previously associated with autism and illustrate the power of using a global approach to study this complex disorder.

The experimental strategy used in the study reported here was designed to tease out differences in gene expression among genetically identical individuals with ASD which might relate to observed differences in the degree of expression of autistic symptoms. Inasmuch as natural variations in gene expression are especially low for monozygotic twins [21,22] (up to 1.76% in the latter study involving 10,000 genes in 5 pairs of monozygotic twins, compared to ~14% in unrelated individuals), such a strategy has been shown to be useful in identifying candidate genes for bipolar disorder [23] and osteoporosis [24]. Moreover, lymphoblastoid cell lines (LCL) derived from blood cells of autistic individuals were used in this study to explore the possibility that biomarkers for autism could be expressed in easily accessible peripheral cells. Indeed, it has been reported previously that LCL from individuals with bipolar disorder displayed altered gene expression in both postmortem brain tissue and lymphoblasts, although one of the genes, LIM, was altered in the opposite direction in LCL [25]. Follow-up genetic association analyses of this gene demonstrated association of a single nucleotide polymorphism with bipolar disorder [26], indicating the usefulness of LCL and DNA microarray analyses in identifying potential biomarkers of a complex neurological disease.

While studies of gene expression in brain tissue may lead to a better understanding of the mechanistic basis for ASD, it is not an appropriate target for diagnostic assays. Ideally, diagnostic assays should use easily obtained patient samples such as blood, although there is no evidence that gene expression or other markers exist in the peripheral blood of ASD patients. However, one may hypothesize that ASD might arise, in part, through dysregulation of expression of specific neuronal genes and that expression differences between affected and unaffected individuals might be present in tissues other than brain. As a test of this hypothesis, we chose to use DNA microarray analysis to examine gene expression in LCL derived from peripheral blood lymphocytes.

Here we report the first study using a genome-scale approach to identify biomarkers for autism. We demonstrate by gene expression profiling on DNA microarrays that: 1) LCL derived from five monozygotic twin pairs discordant for diagnosed autism and/or language impairment show differential gene expression; 2) a number of the most differentially expressed genes are present in pathways critical to the development and function of the nervous system; 3) there appears to be a quantitative relationship between the severity of the autistic phenotype exhibited by the twins and the expression level of certain genes relative to that of the respective genes in cell lines from non-affected siblings; and 4) approximately half of the most highly differentially expressed genes map *in silico *to previously reported chromosomal regions containing autism susceptibility genes or quantitative trait loci.

## Results

### Differential gene expression in lymphoblastoid cell lines from monozygotic twins discordant for classic autism

To determine whether LCL derived from individuals with autism exhibit patterns of gene expression that may be relevant to autism spectrum disorders (ASD), gene expression profiling was performed on LCL derived from 3 sets of male monozygotic twins, one of each pair who met standard diagnostic criteria for autism based on the ADI-R. In each case, the other twin, while not clinically autistic, exhibited autistic traits and was classified either as "broad spectrum" or "not quite autistic" according to guidelines described by the Autism Genetic Resource Exchange (AGRE) repository. Two of the three twin pairs had an unaffected sibling and these were also used for comparison with their respective twin siblings. All of these assays employed an experimental design in which RNA from twin siblings were cohybridized on two-color spotted microarrays containing 39,936 human cDNA elements. Each microarray experiment involved dye-reversal replicates, and was performed in duplicate or, in one case, triplicate for the different sets of twins. The mean log_2_ratios of each gene from the dye-reversal replicates were used for statistical analyses of the biological replicates.

Principal components analysis (PCA) of the combined microarray data with respect to samples from the 3 discordant twin sets showed that genotype is responsible for the major portion of the variation in differential gene expression, reflecting the expected transcriptome heterogeneity among unrelated individuals (see Additional file 1). The microarray data from the 3 sets of discordant twins was analyzed using SAM in order to identify genes that were significantly different from log_2 _= 0 across the biological replicates (n = 3). Twelve hundred genes were identified as significant with an FDR of 26%. Twenty-five genes were found to be up-regulated at least 1.5-fold in the more severely affected twin relative to the other twin (log_2_(ratio) = 0.58) and 19 genes were down-regulated by at least 1.5-fold (Table 1). Of these, eight of the 26 known genes (representing six unique genes) correspond to genes involved in neurological development, function, or disease. Because of this surprising finding, we used quantitative RT-PCR (qPCR) to confirm the differential expression of these specific genes (identified by boldtype in Table 1), as well as several with no known neuronal functions. As shown in Table 1, qPCR confirmed the relative expression levels of all but one of the tested genes, including neurologically relevant argininosuccinate synthetase (ASS), cell death-associated kinase (DAPK1), 5-lipoxygenase-activating protein (ALOX5AP or FLAP), interleukin-6 signal transducer (IL6ST), and Roundabout homolog 1 precursor (ROBO-1), as well as 3 of the "non-neuronal" genes including clotting factor XIIIa (F13A1), eukaryotic translation initiation factor 2C (EIF2C,2), and SAM domain, SH3 domain and nuclear localization signals,1 (SAMSN1).

**Table 1:** Significant up- and down-regulated genes from SAM analysis of microarray experiments on 3 sets of monozygotic twins discordant for autism diagnosis with log2(ratio) ≥ ± 0.58

**Genbank#**	**Gene name or description**	Mean log2(ratio)*	**qPCR^¥^**
	**Upregulated (log2(ratio) ≥ 0.58)**		
R45254	Unknown protein	1.19	
AA448599	F13A1, clotting factor XIIIa precursor	1.08	1.05
AA676466	**ASS**, argininosuccinate synthetase (aa 1–412)	0.92	
AA992985	Unknown protein	0.88	
AA676405	**ASS**, argininosuccinate synthetase (aa 1–412)	0.85	1.47
W07099	**NAGLU**, N-acetylglucosaminidase, alpha	0.85	-0.28
AA044267	P2X5a	0.80	
T49652	**FLAP, ALOX5AP**	0.79	1.01
H57830	histone H1(0) (aa 1–194)	0.78	
R00276	CD38 alt	0.76	
AA488070	Unknown protein	0.75	
W69399	histone H1(0) (aa 1–194)	0.74	
H09567	PAG1	0.74	
AA609189	Unknown protein	0.73	
H02307	**FLAP, ALOX5AP**	0.70	
N50114	PAG1	0.69	
AI091671	Unknown protein	0.67	
N70181	PLEKHG1	0.66	
H24011	Homeodomain-like protein	0.64	
AA412520	Unknown protein	0.63	
AA521362	CR2 receptor	0.62	
AI371096	**DAPK1**, death-associated protein kinase 1	0.62	0.65
T61343	**IL6ST**, IL6 signal tranducer, gp130	0.59	0.58
N29918	ZBTB10	0.59	
T90067	EIF2C2	0.59	0.99
			
	**Downregulated (log2(ratio) ≤ -0.58)**		
T67053	IGLC2	-2.39	
W73790	IGLL1	-2.00	
H18423	Unknown protein	-1.98	
AA448157	CYP1B1	-0.95	
AA644099	Unknown protein	-0.89	
AA933744	ECAT11	-0.84	
AI018127	Unknown protein	-0.83	
AA451886	CYP1B1	-0.77	
AA682565	Unknown protein from neuroblastoma	-0.72	
AI223429	Unknown protein	-0.69	
AA450353	ELMOD1	-0.69	
AA873578	IGHG1	-0.67	
R33402	SAMSN1	-0.67	-0.61
AA173755	**ROBO1**, roundabout 1	-0.66	-0.93
AA022886	retinal degeneration B beta	-0.64	
AA063573	SAMSN1	-0.64	
H99699	mitochondrial aconitase	-0.63	
AI290663	CYBASC3	-0.60	
AA449333	Rab22b	-0.58	

*Mean log_2_(ratio) of gene expression in lymphoblastoid cell lines from children exhibiting classic autism to cell lines from less affected monozygotic twin sibs. SAM analysis revealed 1200 significant genes with an FDR of 26.4%. Only genes for which microarray data is available for all 3 sets of twins are included in this table.

**^¥^**Mean log_2_(ratio) of qPCR data from 3 sets of monozygotic twins. Gene expression was analyzed in triplicate assays (or duplicate for F13A1, EIF2C2, and SAMSN1) and the mean log_2_(ratio) for each respective gene was averaged among the 3 sets of twins. Only one gene, NAGLU, out of the 9 tested from this table was not confirmed by qPCR, possibly because of suboptimal choice of primers for qPCR.

Genes in **boldface type **have been shown to be relevant to neurological development, structure, or function (See Table 3).

Moreover, when the expression profile of cells from the autistic twin was directly compared against that of his respective normal sibling in a dye-reversal microarray experiment, neurologically relevant genes represented 3 of the top 5 most differentially expressed genes (Table 2). Interestingly, the mean log_2_(ratio) of each of these, ASS, CHL1, and FLAP, are higher for the autistic twin than for the more mildly affected twin when each is compared against their respective normal sibling, suggesting a quantitative relationship between differential gene expression (relative to normal individuals) and severity of autistic symptoms, at least for these specific genes. These quantitative differences have also been confirmed by qPCR analyses.

**Table 2:** Expression of ASS, CHL1, and FLAP in 2 sets of discordant monozygotic twins relative to expression levels in their respective normal siblings

**Gene name**	**Genbank #**	**A1**	**M1**	**A2**	**M2**
ASS	AA676405	0.65 (0.81)*	-0.13 (0.27)	1.77 (3.04)	0.18 (0.97)
CHL1	H15267	1.60 (1.64)	0.67 (1.39)	1.15 (0.78)	0.95 (0.60)
FLAP	T49652	0.71 (1.10)	0.13 (0.77)	1.40 (1.45)	-0.09 (-0.39)

Values are mean log_2_(ratio) measures of gene expression between a twin and his respective normal sibling, obtained by DNA microarray analyses with dye reversal replicates

* Values in parentheses are mean log_2_(ratio) measures obtained by triplicate qPCR analyses.

A = autistic twin as diagnosed by ADI-R scoresheet

M = more mildly affected twin who did not meet ADI-R criteria for autism

### Network prediction analysis shows interconnected pathways involving differentially expressed, neurologically relevant genes centered around inflammatory mediators

**Figure 1:**
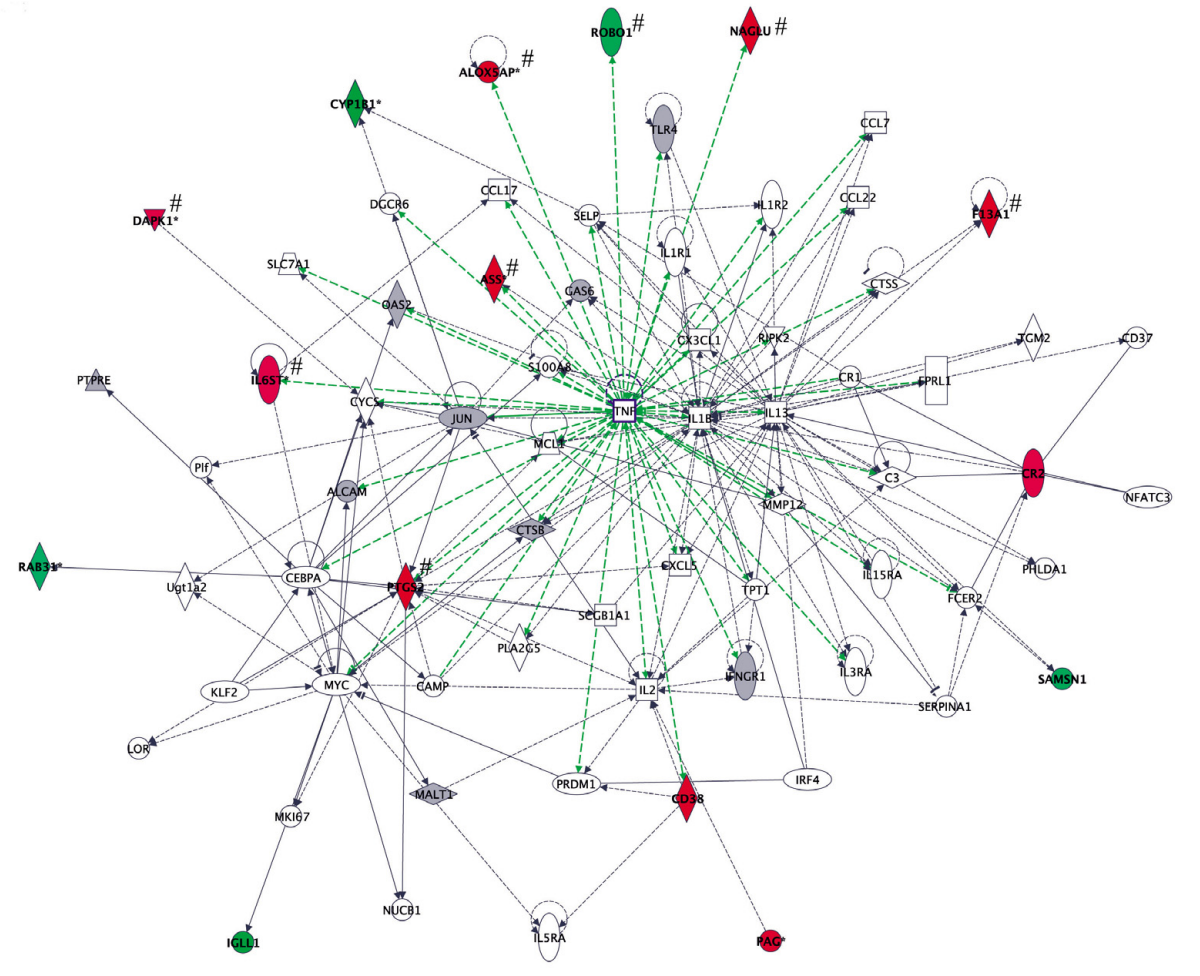
**Gene networks showing inter-relationship between differentially expressed genes in LCL from 3 discordant autistic twin sets using Ingenuity Pathways Analysis software**. The over-expressed (red) and under-expressed (green) genes were identified as significant using SAM analysis (FDR = 26.4%) of microarray data across 3 twin pairs. The log_2 _expression ratio cutoff was set at ± 0.58 and was based upon the *mean *values for each gene. Genes within this network that have a reported role in nervous system development and function are marked with a "#" symbol and include: ASS, ALOX5AP (FLAP), DAPK1, F13A1, IL6ST, NAGLU, PTGS2, and ROBO1. Gray genes are present but do not meet expression cutoff.

Network prediction analysis using Ingenuity Pathways Analysis Software of the 1200 *significant *genes from the SAM analysis further revealed that 25 out of 58 network focus genes exhibiting a differential expression = ± 1.5-fold (i.e., log_2_(ratio) greater than ± 0.58) in at least one discordant twin set are involved in neurological function or disease (Table 3). This expression cutoff was selected because of reports in the literature suggesting that 1.47-fold increases or decreases in gene expression are generally reproducible when Lowess normalization is used [27], and our own ability to confirm expression changes of at least 1.5-fold by qPCR. Of particular note is the gene network that is derived from pathway analysis of the mean expression values (with log_2 _ratio ≥ ± 0.58) across 3 sets of discordant twins which shows that the majority of significantly differentially expressed genes are part of an extended network centered on TNF and other inflammatory mediators (Fig. 1). The neurological functions of 8 of the genes in this network are described in Table 3, which lists all of the network focus genes that exceed the differential expression cutoff (1.5-fold increase or decrease) in at least 1 pair of twins. In comparing this list to that of Table 1, it is clear that, aside from overlapping differentially expressed genes across the 3 sets of twins, there are network genes that are uniquely up- or down-regulated within each of the twin pairs, as illustrated by Additional file 2. The collective data from the above-mentioned microarray and pathway analyses suggested a short list of *novel *candidate autism susceptibility genes with reported neurological functions for further evaluation.

**Table 3:** Network focus genes from Ingenuity Pathways Analysis meeting differential expression cutoff of log2(ratio) ≥ ± 0.58 (1.5-fold increase or decrease) in at least 1 set of twins.

**GenBank #**	**Gene**	Neurological function or disease*
Upregulated		
T49652	**ALOX5AP**	neuronal signaling; possibly neurodegenerative diseases
AA991590	APOC1	
AA147170	**ALS4**	ataxia-ocular apraxia
AA676466	**ASS**	involved in nitric oxide production
H21041	**ATF3**	extension of neurites
AA702350	**AUTS2**	Asperger's syndrome
AI341427	BCAT1	
AA430367	CBS	
R00276	CD38	
AA283949	CDC14A	
N67039	CDK6	
H15267	**CHL1**	extension of neurites; organization of mossy fibers
AA521362	CR2	
AA884403	**CTF1**	myelination, differentiation of neurons
AI371096	**DAPK1**	apoptosis of hippocampal neurons
W00789	**DST**	coalignment of neurofilaments, projection of axons; dysmyelination
AA448599	**F13A1**	stroke
AA149640	**FLT1**	VEGF-induced release of nitric oxide
AA070902	GGA2	
AI375302	**HMGB1**	extension of neurites
AI539460	IL7	
AA406546	**IL6ST**	myelination, development of motor neurons, retraction of dendrites
H09062	MLSTD1	
W07099	**NAGLU**	neurogenesis; vacuolation of neurons
AA598611	**NR4A2**	neurogenesis; metabolism of dopamine
AA707195	**NTRK2**	survival of Purkinje cells; apoptosis of neurons
AA044267	P2RX5	
H09567	**PAG**	possible role in chronic neuroinflammation
AA972337	PAWR	
AA489629	PBEF1	
AI016039	PLXNB2	
R80217	**PTGS2**	activation of astrocytes; spatial memory in mice; apoptosis of neurons
AA495950	RRM2B	
R27457	SLC38A2	
AI091460	SOS1	
N63153	SPRED1	
AI040821	TERE1	
AA970358	TSLP	
Downregulated		
AA779727	**ADAM19**	development of septum
R01732	AMPD3	
AA478589	**APOE**	quantity/morphology of neurons; neurite extension; learning in mice
AA984646	C7orf2	
AA448157	CYP1B1	
AA446027	**EGR2**	myelination; development of motor neurons; routing of axons
AA149096	HCK	
AA620511	HSPA8	
W73790	IGLL1	
AI380522	**ITGB7**	chronic demyelinating disease
AA679503	**KIF1B**	morphology and size of brain; neuron survival
AA029283	LARGE	
T83159	LSP1	
AI351740	**LTB**	neurological disorder in rats
AA022886	PITPNC1	
AI126054	PTK2	
AA173755	**ROBO1**	axon guidance
AA457700	**SCD**	neural regeneration
AA504211	TNFSF11	
N68465	UAP1	

Dataset included 1200 significant genes identified by SAM across three twin sets. FDR was 26.4%.

Genes in **Boldfaced **type are ones of neurological relevance.

*Descriptions of neurological functions were obtained from the Ingenuity Pathways Knowledge Base.

### Differential expression of autism candidate genes in "concordant" autistic twins

Expression analysis of autism candidate genes in LCL from two sets of twins in which *both *individuals were *diagnosed as autistic *surprisingly showed differential expression of several of the candidate genes (Table 4). However, in each case, the "concordant" autistic twin siblings were found to be discordant with respect to severity of language impairment based on each twin's scores on the Peabody Picture Vocabulary Test (PPVT) (see Additional file 3 for profile of subjects studied). Thus, when microarray data from the more language-impaired twin (lower percentile PPVT score) was compared relative to the less impaired twin, the differential gene expression profile overlapped with that which was obtained from the "discordant" twin sets. This result underscores the importance of considering autistic phenotype and/or severity as a means of reducing heterogeneity of gene expression in the search for biomarkers of autism. Interestingly, as shown in Table 4, expression analysis of the candidate genes in cells from monozygotic *nonautistic *twins demonstrated that two of the genes, CHL1 and possibly ROBO1, were differentially expressed. However, it is worth noting that this set of "normal" twins has two autistic siblings. Thus, it is not clear to what extent their gene expression profiles are "normal" since autism exhibits strong genetic influences. It is therefore possible that the differential expression of these two neurologically relevant genes is not coincidental, but does not, by itself, meet the threshold for association with an autistic phenotype. Alternatively, this result might suggest that these genes are not involved in autism. Clearly, this observation on only one set of normal twins warrants future investigation preferably with twins with no autism in their family background. At this time it is difficult to obtain normal monozygotic twins with no autistic siblings from the AGRE repository which focuses exclusively on collecting samples from pedigrees with familial autism.

**Table 4:** Relative expression of candidate genes in monozygotic "concordant" twin pairs with differential language impairment (PPVT percentile scores) and in normal twins

**Candidate Gene**	**Genbank #**	PPVT – 30/42*	**PPVT – 0.1/1**	**Discordant twins^¥^**	**Normal twins**
ASS	AA676405	0.03 (-0.26)^#^	-0.01 (-0.69)	0.85 (1.47)	0.24 (0.54)
CHL1	H15267	1.83 (1.48)	1.99 (1.29)	0.56 (0.46)	1.40 (1.45)
IL6ST	T61343	0.88 (1.33)	0.28 (-0.26)	0.59 (0.58)	0.37 (0.23)
IL6ST	AA406546	1.02 (0.85)	0.34 (-0.14)	0.58 (0.61)	0.43 (0.47)
DAPK1	AI371096	-0.56 (-0.92)	-0.49 (-1.05)	0.62 (0.65)	-0.13 (-0.18)
FLAP	T49652	1.18 (1.20)	0.28 (-0.25)	0.79 (0.58)	-0.19 (-0.34)
ITGB7	AI380522	-1.12 (-1.13)	-0.20 (-0.92)	-0.56 (-0.76)	0.15 (0.04)
EGR2	AA446027	-2.02 (-3.10)	-1.26 (-2.16)	-0.40 (-0.79)	-0.23 (-0.37)
ROBO1	AA173755	-0.13 (0.25)	0.41 (-0.18)	-0.66 (-0.93)	-0.45 (-0.80)

5-HTT^¶^	BC069484	NP^¶ ^(-2.39)	NP (-0.42)	NP (-0.96)	NP (-0.02)

*Values represent the mean log2(ratio) of gene expression from DNA microarray data from 2 sets of monozygotic autistic twins who both met criteria for autism by either ADOS or ADI-R diagnostic tests, but have differential language impairment as indicated by their respective PPVT percentile scores. Data from 2 separate dye-reversal microarray experiments were averaged for each twin set. For each pair of twins, microarray data from the twin with the lower PPVT score was used as the numerator in calculating the gene expression ratio. PPVT – 30/42 refers to the twin pair whose PPVT percentile scores are 30 and 42, while PPVT – 0.1/1 refers to the twin pair whose percentile scores are 0.1 and 1. Interestingly, the two sets of twins share the same mother. The PPVT- 30/42 set are Caucasian males, as are the 3 sets of discordant twins, while the more severely language-impaired twins (PPVT -0.1/1) are black, Latino males.

^#^Values in parentheses are mean log_2_(ratio) expression measures obtained by triplicate qPCR analyses.

^¥^Mean expression value across 3 sets of twins discordant for diagnosis of classic autism.

^¶^Not present (NP) on microarray

### The serotonin transporter (5-HTT) gene is also differentially expressed in lymphoblastoid cells from monozygotic twins discordant in severity of autism and/or language impairment

To evaluate whether differential expression of the serotonin transporter (5-HTT), which is strongly implicated in autism, can be detected in LCL from the autistic and nonautistic twins, qPCR analyses were performed, as 5-HTT is not represented on the microarray platform. Results indicated that, while there is no difference in 5-HTT expression between the nonautistic twins, there is a significant decrease in expression in the more severely affected twin in all of the autistic twin pairs studied, as shown in Table 4. Reduced expression of 5-HTT in blood-derived cells may explain hyperserotonemia in a subset of autistic individuals [28]. It should also be noted that a polymorphism in the promoter region of 5-HTT which results in reduced transcription of 5-HTT is a factor in anxiety-related traits [29,30], common in autism. The present finding suggests that LCL, or their precursor blood lymphocytes, may be useful as reporter cells to evaluate neurologically relevant gene expression differences between autistic and normal individuals.

### Network and global functional analyses of the pooled microarray data on monozygotic twins with autism highlight genes involved in nervous system development and function

**Figure 2:**
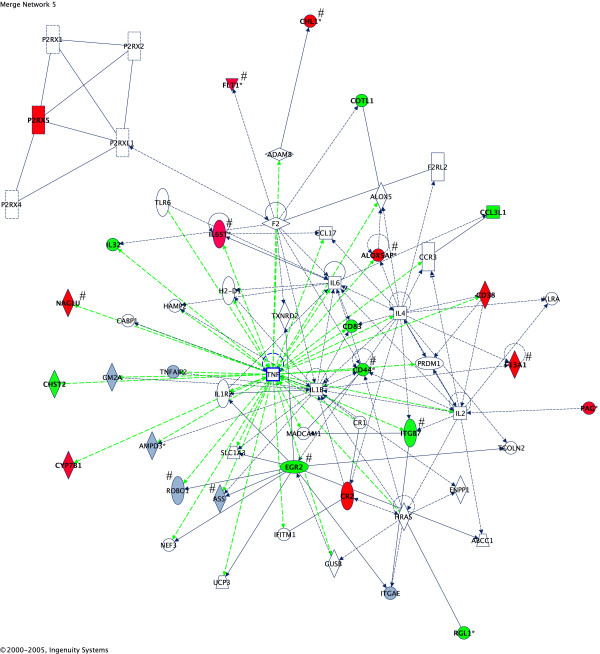
**Gene networks showing inter-relationship between differentially expressed genes in lymphoblastoid cell lines from monozygotic twins discordant in severity of autism spectrum disorder and/or language impairment**. The over-expressed (red) and under-expressed (green) genes were identified as significant using SAM analysis (FDR = 15.6%) of microarray data across 5 twin pairs. The log_2 _expression ratio cutoff was set at ± 0.58 and was based upon the *mean *values for each gene. Differentially expressed genes within this network that have a reported role in nervous system development and function are marked with a "#" symbol and include: ALOX5AP (FLAP), CD44, CHL1, EGR2, F13A1, FLT1, IL6ST, ITGB7, and NAGLU. Gray genes are present but do not meet expression cutoff.

Because of the observed relationship between severity of symptoms and differential expression of candidate genes across the 5 autistic twin pairs studied, SAM was applied to microarray data from all 5 sets of twins to identify genes that were significantly up- or down-regulated across all twin pairs, each pair of which exhibited differential severity with respect to language ability (Table 5). Once again, pathway analysis of the differentially expressed significant genes revealed an extended network centered on TNF and other cytokines (including IL1B, IL4, and IL6, which was highly expressed in the brain tissues of autistic individuals [34]), connecting a number of neurologically relevant genes (Fig. 2). Global functional analysis of the 1281 significant differentially expressed genes from 5 pairs of twins further shows that genes related to "nervous system development and function" are among the most statistically significant, enriched genes across the 5 sets of twins (Table 6).

**Table 5:** Significant genes exceeding expression cutoff across 5 sets of twins with ASD

**Genbank#**	**Gene name or description**	Mean log_2 _(ratio)*
	**Upregulated (log2(ratio) ≥ 0.58)**	
AA448599	F13A1, clotting factor XIIIa precursor	1.50
H15267	**CHL1**, neural cell adhesion molecule	1.10
AA521362	CR2 receptor	1.07
R00276	CD38 alt	0.83
W07099	**NAGLU**, N-acetylglucosaminidase, alpha	0.77
T49652	**FLAP, ALOX5AP**	0.77
AA044267	P2X5a	0.76
R40400	**CHL1**, neural cell adhesion molecule	0.75
H09567	**PAG1**	0.71
AI400399	CYP7B1	0.70
AA149640	**FLT1**	0.67
H17800	Unknown protein	0.67
H02307	**FLAP**, **ALOX5AP**	0.67
AA917693	Unknown protein	0.66
AI017382	ATXN7L1	0.66
AI091671	Unknown protein	0.65
N50114	**PAG1**	0.65
H95977	**Nmd protein**, **PLA1A**	0.65
AA040389	Unknown protein	0.64
H24011	Homeodomain-like protein	0.64
AI275120	Unknown protein	0.63
AA708955	SCHIP1, schwannomin interacting protein 1	0.62
AA406546	**IL6ST**, IL6 signal transducer, gp130	0.62
R79082	PTPRK	0.59
AI241341	**CHL1**, neural cell adhesion molecule	0.59
T61343	**IL6ST**, IL6 signal transducer, gp130	0.59
		
	**Downregulated (log2(ratio) ≤ -0.58)**	
AA446027	**EGR2**, Krox-20 homolog	-0.90
AA630734	seryl-tRNA synthetase	-0.86
R47893	CCL3L1	-0.80
AA682565	Unknown protein from neuroblastoma	-0.76
R78530	COTL1	-0.73
AA933744	ECAT11	-0.73
N58443	GPR55	-0.68
H99699	mitochondrial aconitase	-0.64
H03494	**CD44**	-0.63
AA450353	ELMOD1	-0.63
AA458965	IL32, natural killer cell protein, transcript 4	-0.63
R33402	SAMSN1	-0.62
AA111969	CD83 antigen	-0.60
AI380522	**ITGB7**, integrin beta-7 subunit	-0.60
AA682637	CHST2	-0.59

*Mean log_2_(ratio)of gene expression across 5 sets of twins with ASD. SAM analysis revealed 1281 significant genes with a median FDR of 15.6%. Only genes for which microarray data is available for all 3 sets of twins are included in this table. Genes in **boldface type **have been shown to be relevant to neurological development, structure, or function.

**Table 6:** Global functional analysis: Enrichment of high level functions represented in datasets of differentially expressed genes across 5 sets of monozygotic twins

**High level function**	**Significance^◆ ^(× 10^2^) of enrichment of top five high level functions**				
	
	**Twin sets**				
	
	**361/360**	**809/810**	**2369/2368**	**2595/2596**	**2597/2598**
**Nervous system development and function**	0.008–3.85 (6/19)*	0.12–2.55 (3/7)	0.81–4.79 (5/33)	0.12–4.39 (12/50)	0.02–4.53 (7/26)
**Tissue morphology**	0.008–4.27 (8/19)	NA^¶ ^(0/7)	0.81–4.79 (8/33)	0.08–4.38 (18/50)	0.51–4.03 (4/26)
**Cell death**	0.01–4.27 (8/19)	3.74 (1/7)	0.09–4.79 (4/33)	0.18–4.65 (12/50)	0.09–4.53 (6/26)
**Cellular development**	0.01–4.27 (6/19)	NA (0/7)	0.81–4.79 (4/33)	0.12–4.39 (8/50)	0.03–3.54 (6/26)
**Immune and lymphatic system development and function**	0.03–3.85 (9/19)	NA (0/7)	0.81–4.79 (10/33)	0.33–4.39 (17/50)	0.19–4.03 (9/26)

Global functional analysis of differential gene expression *across 5 sets of monozygotic autistic twins *(each pair identified by blood sample numbers (eg., 361/360) who are discordant with respect to severity of autism or language impairment) was performed using Ingenuity's Pathways Analysis Software.

^◆ ^Significance calculated for each function is an indicator of the likelihood that the high level function is associated with the dataset by random chance. The *p*-value, which is calculated using the right-tailed Fisher's Exact Test, compares the number of user-specified genes of interest (in this case, differentially expressed genes with a log_2_(ratio) cutoff ≥ ± 0.58) that participate in a given function or pathway to the total number of occurrences of these genes in all functional/pathway annotations stored in the Ingenuity Pathways Knowledge Base. It is noteworthy that *genes related to nervous system development and function rank first among the top 5 out of 74 high level functions identified in lymphoblastoid cell lines *on the basis of differentially expressed genes across 5 sets of twins with autism spectrum disorders. The range of significance values for each high level function relates to the different significance values for specific subfunctions within the category.

*(number of differentially expressed genes in functional category/total number of differentially expressed network focus genes with identifiable "high level function" across 5 datasets)

^¶ ^NA: no significance value for this function within the dataset for this pair of twins

### *In silico *mapping of differentially expressed genes to chromosomal regions containing autism candidate genes or quantitative trait loci (QTL)

Although most of the differentially expressed genes identified in this study are novel candidate genes with respect to autism, Table 7 shows that 6 out of 8 of the candidate genes listed in Table 4 [and approximately half (55%) of the differentially expressed genes listed in Tables 3 and 5 (see Additional file 4)] map within or close to chromosomal regions containing previously reported autism candidate genes (ACG) or recently identified QTL for language and nonverbal communication. Interestingly, as observed in Additional File 4 and Table 7 respectively, 32–50% of the genes mapped *in silico *to autism susceptibility or quantitative trait loci are located within language QTL recently identified by Alarcon et al [59], perhaps reflecting the differential severity of language impairment between the co-twins in this study. This observation suggests that the overlay of expression data onto genetic data allows one to focus further genetic and functional analyses on *expressed, neurologically relevant genes that may relate to the behavioral phenotype*. Taken together with the network and global functional analyses described above, these results suggest that blood-derived cells may be useful as surrogates to screen for biomarkers for autism.

**Table 7:** Differentially expressed candidate genes from microarray experiments mapped *in silico *to autism susceptibility genes and QTL

**Candidate gene**	**Genbank #**	**Physical Location**	Reported closely mapped autism candidate genes or QTL*	**Ref**
ASS	AA676405	chr9 (130,349,862–130,406,214)	dopamine beta-hydroxylase (9q34)	77
CHL1	H15267	chr3 (423,533–426,095)	KIAA0121 (3p25.2)	75
IL6R-beta, gp130	T61343	chr5 (55,267,950–55,272,766)	Language QTL chr5:40(0–67)	76
IL6ST	AA406546	chr5 (55,271,809–55,272,305)	Language QTL chr5:40(0–67)	76
DAPK1	AI371096	chr9 (87,552,642–87,553,099)		
FLAP, ALOX5AP	T49652	chr13 (30,207,643–30,236,962)	AUTS3 (13q14-22), HTR2A-2 (serotonin recep. 2A) (13q14-21)	71, 72
ITGB7	AI380522	chr12 (51,871,361–51,887,333)	arginine vasopressin receptor 1A (12q14-15)	69
EGR2	AA446027	chr10 (64,241,755–64,246,081)	Language QTL chr10:107(72–126); HTR-7	76
ROBO1	AA173755	chr3 (78,729,082–78,729,496)		

## Discussion

These studies provide a novel approach to determining candidate genes for autism through the use of peripheral cell lines derived from individuals with ASD. The observations represent a model for the development of a diagnostic screen for autism based on biomarker detection in blood, which is an easily accessible tissue.

In this study, DNA microarrays containing ~40 K human cDNA probes were utilized to examine differences in gene expression profiles in LCL derived from 5 pairs of monozygotic twins with ASD. Three sets of twins were discordant with respect to clinical diagnosis of autism, and 2 sets (with both co-twins diagnosed as autistic) differed with respect to severity of language impairment. We specifically chose this experimental model (direct comparison of identical twins) because differential gene expression in blood leukocytes from monozygotic twins has been reported to be much more restricted than between unrelated controls and, furthermore, the differentially expressed genes exhibited "random variations", showing no specific preference for any functional class [22]. The most remarkable finding of this study is that global functional analysis of the significant differentially expressed genes in LCL from these 5 sets of twins identifies "Nervous system development and function" as a top "high level function" that is significantly enriched across the 5 gene expression datasets (Table 6). Moreover, *in silico *mapping of our most differentially expressed genes across as well as within the twin sets demonstrates that many of these genes are located in or close to chromosomal regions previously identified as autism susceptibility loci by genetic analyses (Table 7 and Additional file 4). Quantitative RT-PCR analysis has further confirmed the differential expression of a subset of our novel candidate genes in the majority of twin sets studied.

Several of these candidate genes and their associated gene networks may provide insight into potential mechanisms involved in the autistic phenotype(s). One of the striking results of the pathway analyses is that a relatively large number of the differentially expressed, neurologically relevant genes are linked in networks that are centered on genes involved in inflammation (see Figs. 1 and 2). The network genes with reported neurological functions include the proteins ASS, ALOX5AP (FLAP), CD44, CHL1, DAPK1, EGR2, F13A1, FLT1, IL6ST, NAGLU, PTGS2, and ROBO1 (See Table 3). The protein ASS regulates the rate-limiting step involved in nitric oxide (NO) production through regeneration of arginine from citrulline, a byproduct of the nitric oxide synthetase (NOS) reaction [31]. Since NO is a major signaling molecule in the brain that has been implicated in several psychiatric disorders, including autism [32], the increased expression of ASS may be of potential relevance to the autistic phenotype. ASS has also been shown to be induced in a rat model of brain inflammation [33], which would be consistent with the hypothesis that neural inflammation may play a role in autism [34]. DAPK1, a cell death-associated serine/threonine kinase which is involved in suppression of integrin activity and disruption of matrix survival signals [35], is also induced by inflammation [36]. Interestingly, the expression of FLT1 (VEGF receptor 1) is also regulated by inflammatory cytokines as well as by NO [37]. Furthermore, the fact that IL6ST (gp130) is increased in LCL from the more severely affected twin, may complement previous observations that IL-6 is the most elevated inflammatory cytokine in the middle frontal gyrus and anterior cingulate gyrus of brain autopsy tissue from autistic individuals [34]. While upregulation of ASS, DAPK1, FLT1, and IL6ST may be responses to inflammation, ALOX5AP (FLAP) and PTGS2 (COX-2) mediate inflammation through the production of leukotrienes [38] and prostaglandins [39]. Interestingly, 5-lipoxygenase, the target of FLAP activation, has been implicated in aging and neurodegenerative diseases [40], as well as other psychiatric disorders [41], including anxiety and depression, which are frequently co-morbid conditions of autism, while a COX-2 inhibitor, celecoxib, has been shown to have therapeutic effects in major depression [42], further suggesting a role for inflammatory processes in psychiatric disease. Collectively, the potential involvement of these specific genes that are associated with neurological function and disease and their presence in pathways regulated by inflammatory mediators lend further support to the neural inflammation model for autism [34], which may be also manifested by immune dysfunctions commonly observed in autism [43].

In addition to the possible role of genes involved in inflammation, a review of the gene list in Table 3 suggests several additional recurring biological themes among the differentially expressed genes with neurological functions: neuronal survival, neurite extension/guidance, and myelination. In this regard, altered expression of EGR2, the most down-regulated gene across 5 twin sets, may be particularly significant (See Table 5 and Additional file 2). EGR2 (Krox-20) is a transcription factor involved in the development of the brain and peripheral nervous system, routing of axons, and myelination [44,45]. Some of these functions may be related to EGR2-mediated regulation of ROBO1, which is involved in neuronal differentiation and axon guidance [46,47], and integrin beta-7 (ITGB7) which has been implicated in chronic demyelinating disease [48]. The expression levels of all three of these genes are relatively reduced with increased severity of autism or language impairment (Table 4). The involvement of cell migration in the pathophysiology of autism is also implicated by the altered expression of CHL1, a novel neural cell adhesion molecule that is involved in neurite migration, outgrowth, connectivity, and survival. Deficiency in CHL1 has been shown to be associated with mental and motor impairments as well as with alterations in exploratory and emotional behavior in mice [49,50], characteristics that are often associated with autism. However, the effect of CHL1 overexpression, which we observe to be associated with the more severe phenotype, has yet to be determined. While the function of such neurologically relevant genes in lymphoblastoid cell lines is unknown, there is growing evidence that gene expression is under genetic control in LCL, as well as in other cells, with one study showing that 31% of the differential expression in LCL among unrelated individuals was heritable [21]. Thus, it is reasonable to postulate that hereditary factors that are responsible for the development of the autistic brain might also be manifested in the LCL as differentially expressed genes. If expression of these genes can be shown to be consistently altered in LCL in case-control studies on a larger sample of unrelated individuals, these cells, and by inference their precursor blood lymphocytes, can potentially be used as reporter cells for diagnosis of ASD. While we have focused on differentially expressed genes of neurological relevance in this study, it should be noted that the biomarkers for autism in LCL or lymphocytes need not have specific neurological functions (as we have also detected and confirmed differential expression of "non-neuronal" genes). Given that ASD is most probably a multigene disorder of varying etiology, a biomarker screen for ASD would likely include a panel of genes consistently associated with ASD phenotypes, in which diagnosis for the disorder will depend upon differential expression of a defined percentage of genes within the consensus set.

The observed relationship between differential gene expression and severity of ASD between monozygotic twins suggests a role for epigenetic factors in ASD. A recent report on normal monozygotic twins indicates that epigenetic differences arise over time, increasing with age and with physical separation from each other after birth [45]. Indeed, epigenetic differences between monozygotic twins have been examined as possible causes for discordancy in schizophrenia as well as bipolar disorder [51-53]. Possible epigenetic mechanisms leading to differences in gene expression include differential methylation, differences in histone acetylation, and micro RNA, although there is no available evidence linking any of these to autism at this time. On the other hand, a mutation in a methyl-CpG binding protein, X-linked MeCP2, has been identified as being involved in 80% of all cases of Rett Syndrome [54], a developmental disorder which overlaps ASD, thus implicating the importance of methylation-dependent gene expression in at least this related disorder. Interestingly, though ubiquitously expressed [55], mutated MeCP2 induces a specific neuronal dysfunction, i.e., Rett Syndrome. One could therefore postulate that differential methylation or differential histone acetylation might give rise to differential expression in LCL from monozygotic twins with ASD and test for global changes in methylation or histone acetylation as done by Fraga et al [45], or for specific changes within a given candidate gene. Such epigenetic modifications in turn could be in response to environmental factors, stochastic processes, or immortalization procedures, which can persist even after the modifying stimulus (eg., inflammation) is removed [56]. If present, these differences could be further tested by evaluation of the methylation/acetylation patterns of DNA/histones in primary lymphocytes from monozygotic twins discordant in severity of autism or language impairment within autism which, while interesting, is beyond the scope of this study.

Regardless of origin, the gene expression differences between monozygotic twins who present with differential severity along the autism spectrum or within a specific behavioral domain (eg., language) are potentially useful, not only as biomarkers for ASD, but also as indicators of genes or metabolic/signaling pathways that may contribute to the autistic phenotype. While our short list of candidate genes (Table 4) focuses on genes with known neurological functions that are similarly up- or down-regulated across twin sets affected by ASD, the set of differentially expressed, neurologically relevant genes that are unique to a given twin set may also be important to the determination of a specific autistic phenotype. Indeed, comparison of the pathways represented in the respective datasets of individual twin pairs reveals not only overlapping genes but also neurologically relevant genes that are differentially expressed in only one of the twin pairs (see Additional file 2). Inasmuch as our microarray analyses *directly compared genetically matched individuals *who differ only in degree of expression of autistic symptoms, it is likely that other genes, not identified in our study, also play a role in the pathophysiology of autism. This experimental design possibly explains why the candidate genes identified here are different from those reported by an earlier genomic study [20] which compared autopsy brain tissues from autistic and normal (nonautistic) controls (i.e., case-control studies). On the other hand, it is interesting that many of our novel genes map closely to genetically identified autism susceptibility genes/loci or QTL (Table 7 and Additional file 4).

Aside from identifying novel candidate genes for autism, our study also illustrates the need for phenotype definition or subgrouping according to severity along a specific behavioral domain for biological studies of autism. Specifically, the results show that the differential gene expression profiles of concordantly autistic twins with differential severity of language impairment mirror some of the differences in gene expression which are observed in the twins with discordant diagnosis of autism, who also exhibit differential language deficits. Thus, for case-control studies in which individuals from the general population are compared against unrelated controls, subgrouping the autistic individuals by phenotype or stratifying them according to severity of symptoms may provide more clarity in analyzing biological data. Towards this goal, we have used several different clustering methods commonly used in DNA microarray analyses to divide over 1300 autistic individuals into endophenotypic subgroups (eg., language, nonverbal communication, and savant skills) based on item scores on the ADIR questionnaire (manuscript in preparation). Based on these methods, the twin siblings analyzed in this study, including those who were both diagnosed as autistic, each fall into different phenotypic clusters (unpublished data), with the exception of one set of twins who were discordant in the diagnosis of autism. These "endophenotypic" differences may therefore account for some of the differences in gene expression profiles between the twin siblings (i.e., co-twins) as well as among the different sets of twins. To test the ability of our clustering algorithm to restrict phenotypic and biological heterogeneity, we evaluated the short list of candidate genes in Table 4 by qPCR in an additional set of "concordant" autistic twins in which both co-twins exhibit the severely language-impaired phenotype. Results showed that, for this twin pair, there are no differences in expression of the candidate genes exceeding a log_2_ratio of ± 0.58 (unpublished data).

## Conclusion

In summary, our study indicates that LCL from genetically identical autistic individuals who differ in severity of autistic symptoms and/or language impairment exhibit differential expression of genes relevant to neurological development, structure, and function. Many of these genes map to chromosomal regions previously identified by genetic analyses as harboring autism susceptibility genes or QTL, thus demonstrating the potential of combined genomic-genetic analyses to prioritize autism candidate genes for further genetic and functional analyses. In addition, a quantitative relationship is seen between severity of symptoms and expression of several autism candidate genes when twins with classic autism or with milder autistic traits are compared against their respective normal siblings. The finding that gene expression differences were also observed in cells from twins who were both diagnosed as autistic, but who differed in severity in language deficits, strongly suggests that autistic phenotype as well as severity of symptoms must be considered in gene expression studies on autistic individuals in order to reduce biological heterogeneity due to these factors. Collectively, these studies provide proof-of-principle that LCL (and possibly their precursor peripheral blood cells) may exhibit biomarkers relevant to autism, and further suggest their potential usefulness as reporter cells in developing a diagnostic screen for autism. While it is unlikely that microarray studies on LCL will identify the etiology(ies) of autism, this global approach to gene expression analyses is expected to highlight molecular or pathway defects related to the pathophysiology of the condition which, in turn, can be targeted for drug therapies. Moreover, as opposed to fixed autopsy tissues in which RNA may have degraded, a live cell model can also be used to examine the functional consequences of the genomic alteration(s) and the efficacy of different pharmacological agents in ameliorating the impaired function.

## Methods

### Cell lines and culture conditions

Lymphoblastoid cell lines (LCL) derived from lymphocytes of 5 pairs of monozygotic twins with ASD were obtained from the Autism Genetic Resource Exchange (AGRE; Los Angeles, CA) and cultured in DMEM with 15% fetal bovine serum and 1% penicillin-streptomycin. Cell lines from normal siblings of 2 sets of twins were also obtained for comparison of gene expression profile with that of their respective autistic siblings. In addition, cell lines from a set of non-autistic monozygotic twins were also studied. All LCL were constructed and maintained by the Cell Repository of the Department of Genetics at Rutgers University under contract from AGRE. To minimize differences in gene expression due to culture and sample workup conditions, all samples that underwent direct comparison of gene expression profile were cultured and harvested at the same time (3 days after passaging) using the same medium preparation and RNA isolation reagents.

### Description of individual donors of cell lines

Additional file 3 provides a case description of all of the subjects included in this study. In brief, all of the twin pairs and normal siblings, with the exception of 1 set of twins, were Caucasian males between the ages of 6 and 16 at the time that blood was drawn. The remaining set of twins (age 12) was of mixed race (black, Hispanic) but had the same mother as one of the Caucasian pairs of autistic twins. For 3 sets of twins (designated "discordant" twins), one twin of each pair met standard diagnostic criteria for autism based on the Autism Diagnostic Interview-Revised (ADIR) [13]. In each case, his co-twin, while not clinically autistic, exhibited autistic traits and could be considered to be on the autism spectrum. These co-twins were described either as "Broad spectrum" or "Not quite autistic (NQA)" by the AGRE repository according to criteria established on the basis of ADI-R scores [79]. Gene expression in cell lines from two of these twin pairs were also directly compared against the gene expression profile in cell lines from their respective "normal" sibling. Two of the 5 sets of twins with ASD (designated "concordant" twins) were examples in which *both *co-twins were diagnosed with autism, but who were discordant in severity of language impairment, as indicated by their respective percentile scores on the Peabody Picture Vocabulary Test (PPVT). The Autism Diagnostic Observation Schedule (ADOS) [14] was used to diagnose one of these sets of twins. None of the individuals whose cells were used presented with any co-morbid condition or mental retardation. All of the phenotypic data were obtained through the AGRE databases [79].

### DNA microarray analyses

RNA was isolated from the LCL using TRIzol Reagent (Invitrogen, CA) according to the manufacturer's protocol. The RNA was further purified using Centricon YM-30 columns and tested for purity on RNA 6000 NanoChips using the Agilent 2100 Bioanalyzer. Labeled cDNA was obtained by incorporation of 5-(3-aminoallyl)-2'deoxyuridine-5'-triphosphate (Ambion, TX) during first-strand synthesis, followed by coupling to the ester of cyanine (Cy-3 or Cy-5) (Molecular Probes, OR) as appropriate according to Standard Operating Protocol (SOP) M004 on The Institute for Genomic Research (TIGR) website [80]. For two-color microarray analyses, the Cy5- and Cy3-labeled cDNA from each pair of twins (or twin and normal sib) were co-hybridized using TIGR SOP M005 to spotted microarrays (TIGR 40 K Human Set) containing 39,936 human cDNA probes which were obtained from Research Genetics. Dye reversal (flip-dye) replicates were included in all analyses, and at least 2 sets of replicates were carried out for each pair of monozygotic twins. Gene expression levels were derived from the scanned hybridized arrays using a combination of TIGR SpotFinder, MIDAS, and MeV analysis programs which are all part of the TM4 Microarray Analysis Software Package available at the above-cited website. These programs have all been previously described in detail [57]. Data analyses included normalization using local LOWESS followed by standard deviation regularization across individual subarrays, and flip-dye consistency checking for dye reversal replicates as implemented in MIDAS [27,58]. The SAM (Significance Analysis of Microarrays) module within MeV was used to determine statistical significance of differential gene expression and false discovery rate (FDR) which corrects for multiple testing.

### Quantitative RT-PCR

Total RNA (same preparations used in microarray analyses) was reverse transcribed into cDNA using the iScript cDNA Synthesis Kit (Bio-Rad, Hercules, CA). Briefly, 2 μg of RNA were added to a 40 μl reaction mix containing reaction buffer, magnesium chloride, dNTPs, an optimized blend of random primers and oligo(dT), an RNase inhibitor, and a MMLV RNase H+ reverse transcriptase. The reaction was incubated at 25°C for 5 minutes followed by 42°C for 30 minutes and ending with 85°C for 5 minutes. The cDNA reactions were then diluted to a volume of 100 μl with water. Real-time PCR was carried out either on a 7900 HT Sequence Detection System from Applied Biosystems using the iTaq SYBR Green Supermix with ROX (Bio-Rad, Hercules, CA) or an Applied Biosystems 7300 Real-Time PCR system using Invitrogen's Superscript III Platinum SYBR Green Two-step qRT-PCR Kit with ROX. Gene-specific primers at a final concentration of 200 nM and 1 μl of cDNA templates were combined into 20 μl reaction mixes and run through 40 cycles of PCR. Quantitation was performed using the Universal 18S rRNA primers (Ambion, Austin, TX) with samples normalized to their 18S rRNA standard curves. Forward and reverse primers are described in Additional file 5.

### Network prediction analyses

Lists of differentially expressed genes identified as "significant" by SAM analysis of microarray data across different sets of twins were analyzed using Ingenuity Pathways Analysis (Ingenuity Systems, Inc.), a web-delivered application that enables biologists to discover, visualize and explore therapeutically relevant networks significant to their specific experimental results (e.g., gene expression array data sets). Specifically, a data set containing gene identifiers (in this case, GenBank Accessions) and their corresponding expression values were uploaded as an Excel spreadsheet using the template provided in the application. Each gene identifier was mapped to its corresponding gene object in the Ingenuity Pathways Knowledge Base. The gene list was filtered prior to analysis with Ingenuity by using a log_2_(ratio) cutoff of 0.58. These genes were then used as the starting point for generating biological networks. The networks are displayed graphically as nodes (genes/gene products) and edges (the biological relationships between the nodes). Human, mouse, and rat orthologs of a gene are stored as separate objects in the knowledge base, but are represented as a single node in the network. The intensity of the node color indicates the degree of up- (red) or down- (green) regulation. When networks from different samples are merged (as in Additional file 2), yellow node color denotes overlapping differentially expressed genes from two or more samples. Nodes are displayed using various shapes that represent the functional class of the gene product, as described on Ingenuity's website [81].

### Global functional analyses

Biological functions were assigned to the overall analysis (across data from 5 monozygotic twin pairs) by using the findings that have been extracted from the scientific literature and stored in the Ingenuity Pathways Knowledge Base [81]. The biological functions assigned to the analysis are ranked according to the significance of that biological function to the analysis. A Fisher's exact test is used to calculate a *p*-value determining the probability that the biological function assigned to the analysis is explained by chance alone. Again, a differential expression cutoff of log_2_(ratio) ≥ ± 0.58 was used.

### *In silico *mapping of differentially expressed genes

The physical locations of each of the significant differentially expressed genes with log2(ratio) ≥ ± 0.58 were obtained using TIGR's Resourcerer Gene Annotation Software [80]. These locations were then compared manually to those of autism candidate genes (ACG) or quantitative trait loci (QTL) identified on the basis of published genetic linkage and association studies [59-78]. For comparison, using Human Genome Build 35 and defining genes as the collection annotated by Ensembl (a total of 24974), we have found that the set of non-redundant genes mapping to the large, often overlapping autism susceptibility or QTL regions is 3838, or about 15.4% of the total.

### Submission of microarray data to GEO repository

The GEO accession number for all of the microarray data is GSE4187.

## Abbreviations

ACG: autism candidate genes

ADIR: Autism Diagnostic Interview-Revised

ADOS: Autism Diagnostic Observation Schedule

AGRE: Autism Genetic Resource Exchange

ASD: autism spectrum disorders

ASS: argininosuccinate synthetase

CHL1: cell adhesion molecule with homology to L1CAM

DAPK1: death-associated protein kinase 1

EGR2: early growth response 2 protein (Krox-20)

FLAP: 5-lipoxygenase activating protein

FLT1: Fms-related tyrosine kinase 1 (VEGF receptor)

5-HTT: 5-hydroxytryptamine (serotonin) transporter

ITGB7: integrin beta-7

LCL: lymphoblastoid cell lines

PPVT: Peabody Picture Vocabulary Test

PTGS2: prostaglandin-endoperoxide synthase 2 (cyclooxygenase-2, COX-2)

QTL: quantitative trait loci

ROBO1: roundabout, axon guidance receptor

SAM: Significance Analysis of Microarrays

## Authors' contributions

VWH conceived of the study and experimental design using monozygotic twins discordant in autism. She also carried out the sample preparations, DNA microarray hybridizations, data analyses, and manuscript preparation. JQ and NHL provided helpful advice with regard to microarray data analyses and also contributed to manuscript preparation. BCF and SH were responsible for the confirmatory qRT-PCR analyses. All authors read and approved the final manuscript.

## Supplementary Material

Additional File 1 **Principal components analysis of microarray data from the 5 sets of monozygotic twins with ASD, with each color representing a separate pair of twins**. This figure shows that genotype is a major contributor to variations in overall gene expression profile. Each point on the graph represents a dye-reversal experiment on a given twin pair. Note that even the 2 pairs of twins who share the same mother but have different fathers (pink and yellow points) are distinguishable from each other. Click here for file

Additional File 2 **A representative gene network showing overlap of some neurologically relevant genes among 3 discordant autistic twin sets using Ingenuity Pathways Analysis software**. Genes shown in yellow represent overlap of differentially expressed genes in 2 or more sets of twins, whereas the red and green nodes correspond to genes that are up- or down-regulated, respectively, in only 1 twin set. The expression cutoff was set at log_2_(ratio) = ± 0.58 for each twin set. The 12 genes marked by "#" are known to be involved in nervous system development and function. Click here for file

Additional File 3 **Case description of subjects from whom LCL were derived and used in this study**. (Self-explanatory) Click here for file

Additional File 4 **Differentially expressed genes within or across twin sets mapped within or close to autism candidate genes or quantitative trait loci**. This table shows that many of the differentially expressed genes map *in silico *to autism susceptibility loci or quantitative trait loci identified by genetic analyses. Click here for file

Additional File 5 **Primers used for quantitative RT-PCR analyses**. (Self-explanatory) Click here for file
