# Supplementary figures and images for: Gene expression profiling of lymphoblastoid cell lines from monozygotic twins discordant in severity of autism reveals differential regulation of neurologically relevant genes
Source: BMC Genomics. 2006 May 18;7:118. doi: 10.1186/1471-2164-7-118 (PMC1525191; doi:10.1186/1471-2164-7-118)

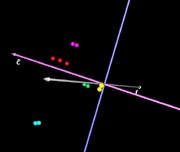

Supplement: Additional File 1 — Principal components analysis of microarray data from the 5 sets of monozygotic twins with ASD, with each color representing a separate pair of twins. This figure shows that genotype is a major contributor to variations in overall gene expression profile. Each point on the graph represents a dye-reversal experiment on a given twin pair. Note that even the 2 pairs of twins who share the same mother but have different fathers (pink and yellow points) are distinguishable from each other. [file 1471-2164-7-118-S1.jpeg]

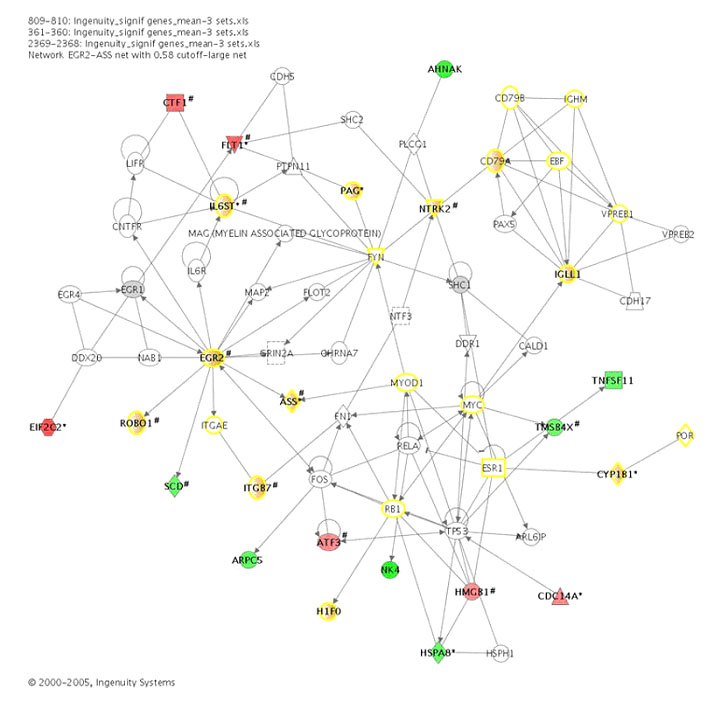

Supplement: Additional File 2 — A representative gene network showing overlap of some neurologically relevant genes among 3 discordant autistic twin sets using Ingenuity Pathways Analysis software. Genes shown in yellow represent overlap of differentially expressed genes in 2 or more sets of twins, whereas the red and green nodes correspond to genes that are up- or down-regulated, respectively, in only 1 twin set. The expression cutoff was set at log2(ratio) = ± 0.58 for each twin set. The 12 genes marked by "#" are known to be involved in nervous system development and function. [file 1471-2164-7-118-S2.jpeg]
