# Supplementary material for: Gene expression profiling of lymphoblastoid cell lines from monozygotic twins discordant in severity of autism reveals differential regulation of neurologically relevant genes
Source: BMC Genomics. 2006 May 18;7:118. doi: 10.1186/1471-2164-7-118 (PMC1525191; doi:10.1186/1471-2164-7-118)
Supplement: Additional File 3 — Case description of subjects from whom LCL were derived and used in this study. (Self-explanatory) [file 1471-2164-7-118-S3.pdf]

Additional file 3. Case description of subjects from whom LCL were derived and used in this study.

Individual ID	Blood ID	Ethnicity	Zygosity	Age*	Status	PPVT (%ile)	Raven
AU002704	HI0361	Caucasian	MZ	8	Autism		108
AU002703	HI0360	Caucasian	MZ	8	Br. Spec.	79 (8)	105
AU057904	HI0809	Caucasian	MZ	6	Autism	35 (0.1)	83
AU057905	HI0810	Caucasian	MZ	6	Br. Spec.	117 (87)	110
AU057903	HI0813	Caucasian		10	nonautistic		
AU0885303	HI2369	Caucasian	MZ	16	Autism	No data	No data
AU0885302	HI2368	Caucasian	MZ	16	NQA	"	"
AU0885304	HI2357	Caucasian		19	nonautistic	"	"
AU0616301	HI2595	Caucasian	MZ	15	Autism[¥]	92 (30)	104
AU0616302	HI2596	Caucasian	MZ	15	Autism[¥]	97 (42)	94
AU0616303	HI2597	Mixed, Hispanic	MZ	12	Autism	40 (< 0.1)	80
AU0616304	HI2598	Mixed, Hispanic	MZ	12	Autism	66 (1)	107
AU1165305	HI2745	Caucasian	MZ	9	nonautistic		
AU1165306	HI2744	Caucasian	MZ	9	nonautistic		

*Age at time of inclusion in study

[¥]Diagnosed with ADOS rather than ADIR

Br. Spec.: Defined as “broad spectrum” by AGRE

NQA: Defined as “not quite autistic” by AGRE
