# Supplementary material for: Gene expression profiling of lymphoblastoid cell lines from monozygotic twins discordant in severity of autism reveals differential regulation of neurologically relevant genes
Source: BMC Genomics. 2006 May 18;7:118. doi: 10.1186/1471-2164-7-118 (PMC1525191; doi:10.1186/1471-2164-7-118)
Supplement: Additional File 4 — Differentially expressed genes within or across twin sets mapped within or close to autism candidate genes or quantitative trait loci. This table shows that many of the differentially expressed genes map in silico to autism susceptibility loci or quantitative trait loci identified by genetic analyses. [file 1471-2164-7-118-S4.pdf]

Add'l file 4_Differentially expressed genes within or across twin sets mapped within or close to autism candidate genes or quantitative trait loci

GenBank #	Gene	Physical map	Band	Reported QTL or ACG*	Map Location$^{\Delta}$	Refs.
H99699	ACO2	chr22:40,249,136-40,249,479	22q13.2			
AA779727	**ADAM19**[¶]	chr5:156,836,890-156,839,743	5q33.3	Language QTL	chr5: 125 (98-152)[¥]	59
T49652	**ALOX5AP**	chr13:30,207,643-30,236,962	13q12.3	AUTS3, HTR2A-2	13q14-q22. 13q14-q21	64, 65
AA147170	**ALS4**	chr9:132,168,835-132,169,190	9q34.13	dopamine beta-hydroxylase	chr9:135,574,213-135,774,497; 9q34	60
R01732	AMPD3	chr11:10,433,286-10,485,260	11p15.4			
AA991590	APOC1	chr19:50,110,006-50,114,439	19q13.31	APOE	19q13.2	66
AA478589	**APOE**	chr19:50,103,783-50,104,362	19q13.31	APOE	19q13.2	
AA676466	**ASS**	chr9:130,384,688-130,405,958	9q34.11	dopamine beta-hydroxylase	chr9:135,574,213-135,774,497; 9q34	60
H21041	**ATF3**	chr1:209,181,152-209,181,560	1q32.3			
AI017382	ATXN7L1	chr7:105,109,492-105,111,716	7q22.3	AUTS 1 on chromosome 7q; RELN	chr7:100,270,094-100,470,368; 7q22	61, 62
AA702350	**AUTS2**	chr7:69,701,671-69,702,124	7q11.22	AUTS2	chr7:69,701,671-69,702,124	68
AI341427	BCAT1	chr12:24,880,649-24,894,152	12p12.1			
AA984646	C7orf2	chr7:156,090,884-156,091,208	7q36.3	EN2	7q36	71
AA430367	CBS	chr21:43,346,369-43,369,493	21q22.3			
R47893	CCL3L1	chr17:31,647,966-31,648,404	17q12	Language QTL	chr17: 45 (13-96)	59
R00276	CD38	chr4:15,456,170-15,527,338	4p15.32	HLN2	4p15.3	73
AA111969	CD83	chr6:14,244,635-14,244,846	6p23			
AA283949	CDC14A	chr1:100,697,433-100,697,853	1p21.2			
N67039	CDK6	chr7:91,879,642-91,880,092	7q21.2	CMT2F	7q11-21	74
H15267	**CHL1**	chr3:274,882-275,218	3p26.3	KIAA0121	3p25.2	61
AA682637	CHST2	chr3:144,324,253-144,324,489	3q23	Language QTL	chr3: 147 (126-170)	59
R78530	COTL1	chr16:83,156,719-83,157,052	16q24.1	Language QTL	chr16: 134 (127) (78)	59
AA521362	CR2	chr1:204,051,081-204,051,632	1q32.2			
AA884403	**CTF1**	chr16:30,822,197-30,822,382	16p11.2	Nonverbal communication QTL	16p12-13	63
AI400399	CYP7B1	chr8:65,671,716-65,679,901	8q12.3			
AI371096	**DAPK1**	chr9:87,552,642-87,553,099	9q21.33			
W00789	**DST**	chr6:56,636,931-56,637,351	6p12.1			
AA933744	ECAT11	chr1:62,388,845-62,389,499	1p31.3			
AA446027	**EGR2**	chr10:64,241,762-64,242,293	10q21.3	Language QTL	chr10: 107 (72-126)	59
AA450353	ELMOD1	chr11:107,042,443-107,042,715	11q22.3			
AA448599	**F13A1**	chr6:6,089,901-6,090,339	6p25.1			
AA149640	**FLT1**	chr13:27,859,860-27,860,230	13q12.3	AUTS3, HTR2A-2	13q14-q22. 13q14-q21	64, 65
AA070902	GGA2	chr16:23,386,296-23,388,914	16p12.1	RP22	16p12.3-p12.1	73
N58443	GPR55	chr2:231,599,868-231,600,296	2q37.1			
AA149096	HCK	chr20:30,145,453-30,153,082	20q11.21			
AI375302	**HMGB1**	chr13:29,930,931-29,931,301	13q12.3	AUTS3, HTR2A-2	13q14-q22. 13q14-q21	64, 65
AA620511	HSPA8	chr11:122,433,412-122,433,823	11q24.1			
W73790	IGLL1	chr22:22,239,869-22,241,827	22q11.23			
AA458965	IL32, NK4	chr16:3,055,381-3,059,572	16p13.3	Language QTL	chr16: 8 (0-20)	59
AA406546	**IL6ST**	chr5:55,271,809-55,272,305	5q11.2	Language QTL	chr5: 40 (0-67)	59
AI539460	IL7	chr8:79,808,141-79,814,872	8q21.12			
AI380522	**ITGB7**	chr12:51,871,374-51,872,549	12q13.13	arginine vasopressin receptor, 1A	12q14-q15	67
AA679503	**KIF1B**	chr1:10,301,980-10,302,404	1p36.22	Language QTL	chr1: 30 (9-41)	59

Add'l file 4_Differentially expressed genes within or across twin sets mapped within or close to autism candidate genes or quantitative trait loci

AA029283	LARGE	chr22:31,993,629-31,994,110	22q12.3			
T83159	LSP1	chr11:1,865,114-1,869,808	11p15.5			
AI351740	**LTB**	chr6:31,656,328-31,656,552	6p21.33			
H09062	MLSTD1	chr12:29,375,639-29,375,879	12p11.22			
W07099	**NAGLU**	chr17:37,948,670-37,949,193	17q21.2	Language QTL; 17q21 fine map	chr17: 45 (13-96)	59, 67
AA917693	NR3C1	chr5:142,768,749-142,794,477	5q31.3	Language QTL	chr5: 125 (98-152)	59
AA598611	**NR4A2**	chr2:157,007,118-157,007,513	2q24.1	cAMP-GEFII, DLX1, DLX2, HOXD1, NE	2q21-q33	70
AA707195	**NTRK2**	chr9:84,655,356-84,655,789	9q21.33			
AA044267	P2RX5	chr17:3,523,268-3,546,395	17p13.2	FIMG1	17p13	73
N50114	**PAG**	chr8:82,048,965-82,049,320	8q21.13			
AA972337	PAWR	chr12:78,488,215-78,488,615	12q21.2			
AA489629	PBEF1	chr7:105,482,685-105,483,083	7q22.3	AUTS 1 on chromosome 7q; RELN	chr7:100,270,094-100,470,368; 7q22	61,62
AA022886	PITPNC1	chr17:63,119,259-63,119,494	17q24.2			
H95977	**PLA1A, Nmd**	chr3:120,819,670-120,830,364	3q13.33	Language QTL	chr3: 147 (126-170)	59
AI016039	PLXNB2	chr22:49,015,865-49,016,311	22q13.33			
R80217	**PTGS2**	chr1:183,372,637-183,372,865	1q31.1	Reanalysis of whole genome scan	chr1:183,000,000	76
AI126054	PTK2	chr8:141,958,441-141,958,919	8q24.3	Nonverbal communication QTL	8q23-24	63
R79082	PTPRK	chr6:128,332,083-128,332,365	6q22.33			
AA173755	**ROBO1**	chr3:78,729,082-78,729,496	3p12.3			
AA495950	RRM2B	chr8:103,285,907-103,286,293	8q22.3	Nonverbal communication QTL	8q23-24	63
AA630734	SARS	chr1:109,491,951-109,492,822	1p13.3	Nonverbal communication QTL	1p13-q12	63
AA457700	**SCD**	chr10:102,112,573-102,112,966	10q24.31	Language QTL	chr10: 107 (72-126)	59
AA708955	SCHIP1	chr3:161,097,412-161,097,852	3q25.33	Language QTL	chr3: 147 (126-170)	59
R27457	SLC38A2	chr12:45,040,635-45,040,927	12q13.11	arginine vasopressin receptor, 1A	12q14-q15	67
AI091460	SOS1	chr7:127,471,196-127,495,720	7q32.1	GMR8	7q31-33	72
AI091671	Spidroin 1	chr15:72,454,484-72,454,905	15q24.1	SLP-1, hUNC-24	15q24-25	77
N63153	SPRED1	chr15:36,433,411-36,433,545	15q14			
AI040821	TERE1	chr1:11,281,557-11,281,945	1p36.22	Language QTL	chr1: 30 (9-41)	59
AA504211	TNFSF11	chr13:42,079,796-42,080,148	13q14.11	AUTS3, HTR2A-2	13q14-q22. 13q14-q21	64, 65
AA970358	TSLP	chr5:110,440,005-110,440,383	5q22.1	Language QTL	chr5: 125 (98-152)¨	59
N68465	UAP1	chr1:159,290,229-159,300,829	1q23.3			
H24011	Unknown	chr7:140,628,082-140,628,452	7q34	OTSC2, Language QTL	7q34-36; chr7:139465749 - 154005714	74, 78
AI275120	Unknown	chr2:149,896,904-149,897,281	2q23.2	cAMP-GEFII, DLX1, DLX2, HOXD1, NE	2q21-q33	70
AA040389	Unknown	chr4:100,348,265-100,348,631	4q23			
AA682565	Unknown	chr4:185,630,597-185,630,964	4q35.1			
H17800	Unknown	chr9:3,208,297-3,208,697	9p24.2			
		¶Genes in **bold** are involved in		*ACG = autism candidate gene	ΔIdentified by genetic analysis	
		nervous system development				
		and function			¥Peak and (region)	
