# Supplementary material for: Gene expression profiling of lymphoblastoid cell lines from monozygotic twins discordant in severity of autism reveals differential regulation of neurologically relevant genes
Source: BMC Genomics. 2006 May 18;7:118. doi: 10.1186/1471-2164-7-118 (PMC1525191; doi:10.1186/1471-2164-7-118)
Supplement: Additional File 5 — Primers used for quantitative RT-PCR analyses. (Self-explanatory) [file 1471-2164-7-118-S5.pdf]

Additional file 5. Primers used for quantitative RT-PCR analyses

Gene Name	GenBank #	Forward Primer (5' to 3')	Reverse Primer (5' to 3')
ASS	AA676405	GAAGTGCGCAAAATCAAACA	CTGCACTTTCCCTTCCACTC
CHL1	H15627	TTTAGATGCACCCGTGTTTG	AGCACACCAACATTTCTCATT
DAPK1	AI371096	CGCTACCTCTCTGTCCCTTG	AGGATTCCCTTCTCCCCTTT
EGR2	AA446027	CCCATCACAGGTTTTTGACC	TCTTTTTGCTGTCCCCACTT
EIF2C2	T90067	GCGTGTCTGTTTGGCTTTG	CCTGCAAGGCAAGGAATG
F13A1	AA448599	CACCCATCTCTCAGGAATCA	CACTAGATCCGCCAGCTTCT
FLAP	T49652	GACGATCTCCACCACCATCT	AGAATGCTCTCAAGAGCTGAA
IL6ST	T61343	TTAAAAGGTGGCAGCTCAGG	TCATCACACGACCCATCAAC
IL6ST	AA406546	GCTGGGCTCATGTAGTTATGG	CATCAGAGTGGCTTAGGGACA
ITGB7	AI380522	CATCACGACCACCATCAATC	CTCCAGTTCCCACTGTCCTC
NAGLU	W07099	TCCAACAGCACCAGTTTGAC	AGCCGGGGTAATATTTGAGG
NAGLU	W07099	ACACTCCGGAGCAGTAGCC	AAAGGACCCAGTGCCAGATT
ROBO1	AA173755	CTGACCCCAGTGGAAAACA	CCCTTAGTACTGCACGCCTTT
SAMSN1	AA063573	AAAGACAAAACATTTCCACAAT	CACATTTTATCCTGATCCACA
5-HTT	BC069484	CCTCCAGCCACTTATTTCCA	ACCTCCATCCACATCCTCAC
18S		CCGCAGCTAGGAATAATGGA	CCCTCTTAATCATGGCCTCA
Control Primers			
MLC1	AA196486	TGAAGAGCTGAATGCCAAGA	CCTTGTCAAAGACACGCAGA
TCRb	AA909476	CATGAGCATCAGCCTTCTGT	GAAAGGCCTGTCCACTCTCC
Werner HIP	AA189052	GGCTATGGCAAAGGCTACAA	GAGTCAGCACCTCCTCTGCT
